# Influencing factors of polyspermy during in vitro fertilization with antagonist protocol

**DOI:** 10.12669/pjms.41.2.9832

**Published:** 2025-02

**Authors:** Hongzhi Shi, Qi Song, Jiajia Liu, Chen Li, Rongrong Liu

**Affiliations:** 1 Hongzhi Shi Department of Reproductive Medicine, Maternity & Child Care Center of Qinhuangdao, Qinhuangdao 066000, Hebei, China; 2 Qi Song Department of Reproductive Medicine, Maternity & Child Care Center of Qinhuangdao, Qinhuangdao 066000, Hebei, China; 3 Jiajia Liu Department of Reproductive Medicine, Maternity & Child Care Center of Qinhuangdao, Qinhuangdao 066000, Hebei, China; 4 Chen Li Department of Reproductive Medicine, Maternity & Child Care Center of Qinhuangdao, Qinhuangdao 066000, Hebei, China; 5 Rongrong Liu Children Protect Branch, Maternity & Child Care Center of Qinhuangdao, Qinhuangdao 066000, Hebei, China

**Keywords:** In vitro fertilization, Polyspermy, Influencing factor, Antagonist

## Abstract

**Objective:**

To explore the influencing factors of polyspermy in long-term fertilization during in vitro fertilization (IVF) with antagonist protocol.

**Methods:**

This was a retrospective case-control study. Patients with secondary infertility undergoing assisted reproductive treatment in the Department of Reproductive Medicine of Maternity & Child Care Center of Qinhuangdao between January 2020 and May 2023 were selected. Five hundred and thirty-six ovulation induction cycles with antagonist protocol were included and divided into the control group (multiple pronuclei(PN) rate= 0%, n=347) and the polyspermy group (multiple PN: 2PN ≥20%, n=189) according to the incidence of polyspermy. The general data and embryonic development of the two groups were statistically analyzed.

**Results:**

There were no statistically significant differences in male age, infertile duration, body mass index (BMI), basal follicle-stimulating hormone (FSH) level, number of follicles with a diameter of ≥16mm, total dose of gonadotropin (Gn), total Gn stimulation days, and sperm concentration between the two groups (all P>0.05). Similarily no statistically significant differences in female age, levels of estradiol (E2), LH, and progesterone (P) and number of follicles with a diameter of ≥14 mm on human chorionic gonadotropin (hCG) injection day, the total number of eggs retrieved, amount of Gn/egg, and fertilization concentration (sperms/egg) were observed between the two groups (all P<0.05). The results of logistic binary regression analysis showed that the level of LH on hCG injection day was an independent risk factor for the incidence of polyspermy in antagonist protocol.

**Conclusion:**

In the process of ovulation induction in antagonist protocol, the appropriate addition of LH can improve embryo outcomes.

## INTRODUCTION

Polyspermy occurs when more than 2PN are observed in a fertilized embryo during in vitro fertilization (IVF). The increase of polyspermic embryos may reduce the number of available embryos and the egg utilization rate. In addition, whether the development potential of all embryos in the cycle with polyspermy will be adversely affected is a matter of great concern.^1^-^4^ Numerous studies on the influencing factors of polyspermy have been reported. Most of these studies were based on the incidence of polyspermy,^5^ and there are also studies in which the number of polyspermic embryos was used for grouping and analysis.^6^ Cycles with a poor fertilization rate may be ignored due to the low incidence of polyspermy, and these cycles, however, is the treatment priority. In the present study, in order not to miss the data of the cycles with a poor fertilization rate, cycles with a high proportion of polyspermic embryos were considered rather than using the total number of eggs as the denominator in the calculation as the traditional method did.

A ratio of multiple PN: 2PN ≥20% was used as a cutoff value for grouping. All cycles with ovulation induction using antagonist protocol and long-term fertilization were selected to reduce the influence of confounding factors and objectively explore the influencing factors of polyspermy in long-term fertilization during in vitro fertilization (IVF) with antagonist protocol, providing experiences for clinical and laboratory work.

## METHODS

This was a retrospective case-control study. Clinical data of patients undergoing in vitro fertilization-embryo transfer (IVF-ET) procedures in Maternity & Child Care Center of Qinhuangdao between January 2020 and May 2023 were retrospectively analyzed. Five hundred and thirty-six ovulation induction cycles with antagonist protocol were included and divided into the control group (n=347) and the polyspermy group (n=189). According to the incidence of polyspermy.

### Ethical Approval:

The study was approved by the Institutional Ethics Committee of Maternity & Child Care Center of Qinhuangdao (No.:QHDFY-2023031008; Date: March 11, 2023), and written informed consent was obtained from all participants.

### Inclusion criteria:

- Using antagonist protocol for ovulation induction.
- With an age of 20~40 years old.
- With a serum basal FSH level of <10 IU/L.
- Using long-term fertilization method.

### Exclusion criteria:

- Completely failed fertilization cycles (absence of both normal and abnormal fertilizations).
- Endometriosis.
- Hydrosalpinx.

### Ovulation induction:

Appropriate ovulation induction protocol was made according to the age and ovarian reserve of the patients. Follicle-stimulating hormone(FSH) was injected on the 2nd to 3rd day of menstruation, and agonist or Ovidrel 200 U was injected according to the reactions of the patient when three follicles with a diameter of ≥18 mm appeared. Eggs were retrieved about 36 hours after the injection.

### Semen preparation and fertilization:

Males were required to keep abstinence for 2-7 days before semen retrieval, and semen was collected by masturbation on the day of retrieval. Semen was processed by density-gradient centrifugation and swim-up method. 40% Pureception and 80% Pureception (Cooper Company, USA) were used as the separation solution in the density-gradient centrifugation, and sperm fertilization solution (ART-1020, Sage, USA) was used as the washing solution and swim-up solution. 38-40 hours after hCG injection, fertilization was carried out in an IVF petri dish. One ml pre-equilibrated fertilization solution was added into the middle of each dish. one dish was used for the fertilization of ≤10 eggs and two dishes for the fertilization of >10 eggs, with a final fertilization concentration of 20,000-30,000 sperms/egg.

### Observation of fertilization status:

Long-term fertilization was conducted and PN was observed 17-19 hours after fertilization. The presence of 2PN in an egg was considered normal fertilization, and the presence of ≥3 PN in an egg was considered polyspermy.

### Embryo culture:

The embryo development was observed 66-70 hours after fertilization, and the blastocyst development was observed 96-112 hours after fertilization. SAGE sequential medium (Sage, USA) was used for embryo culture. Cleavage-stage embryos and blastocysts were evaluated according to consensus.

### Grouping:

Cycles were grouped according to the ratio of multiple PN: 2PN. Cycles with no polyspermy were included in the control group and those with a ratio of multiple PN: 2PN of ≥20% were included in the polyspermy group.

### Outcome measures:

The general data and embryonic development of patients in the control group and the polyspermy group were compared.

### Statistical Analysis:

SPSS23.0 software was used for data analysis. The power of test / confidence interval was 95%. Enumeration data were presented as percentages, and χ^2^ test was used for comparison between groups. Measurement data were presented as n (%) and *χ̅*±*S*, and an independent sample t test was used for comparison between groups. Differences with a p value of <0.05 were considered statistically significant.

## RESULTS

A total of 537 cycles were included in the study, of which 347 cycles were included in the control group, with an incidence rate of polyspermy of 0%, and 189 cycles in the polyspermy group, with a ratio of multiple PN:2PN of ≥20%. There were no statistically significant differences in male age, BMI, infertile duration, basal FSH level, the total dose of Gn, the total number of Gn stimulation days, the number of follicles with a diameter of ≥16 mm, and sperm concentration between the two groups (all P>0.05). Similarily no statistically significant differences in female age, amount of Gn/egg, levels of E2, LH, and P on hCG injection day, number of follicles with a diameter of ≥14 mm on hCG injection day, fertilization concentration(x 10,000 sperms/egg), and total number of eggs retrieved were observed between the two groups (all p<0.05) (Table-I).

**Table-I:** Comparison of basic data between the two groups.

Items	Group A(n=347)	Group B(n=189)	*P*
Female age	33.04±4.07	32.27±3.98	0.034
Male age	33.98±4.56	33.38±4.26	0.136
Female BMI	24.50±4.51	24.53±4.46	0.932
Infertile duration(years)	3.41±2.75	3.52±2.85	0.681
Basal FSH	7.22±3.04	6.28±1.85	0.525
Total dose of Gn	2822.68±778.54	2790.09.78±825.41	0.651
Number of Gn injection days	10.51±1.97	10.69±1.70	0.304
Gn/egg	382.24±266.79	303.02±239.13	0.001
E2 on hCG injection day	9541.13±8497.63	10979.55±9466.29	0.102
LH on hCG injection day	3.55±2.93	2.90±2.03	0.003
P on hCG injection day	2.63±2.15	3.06±2.24	0.030
Number of follicles with a diameter of ≥16mm	5.46±3.31	5.88±3.30	0.159
Number of follicles with a diameter of ≥14mm	7.88±4.91	8.73±4.60	0.053
Average number of eggs retrieved	8.41±6.18	10.21±6.79	0.002
Sperm concentration	20.83±4.05	21.13±3.35	0.348
Fertilization concentration ( x10,000 sperms/egg)	4.20±3.78	3.18±2.35	0.000

In order to reduce the effects of confounding factors, related factors with a p<0.05 in the univariate analysis were included in the logistic regression model for binary regression analysis. The results showed that the level of LH on hCG injection day was an independent risk factor for polyspermy (Table-II). The egg utilization rate was decreased in the polyspermy group compared with that in the control group, and the difference was statistically significant(P<0.05) (Table-III).

**Table-II:** Binary Logistic regression analysis of influencing factors.

		B	Standard error	Wald	Degree of freedom	Significance	Exp(B)	EXP(B) 95% CI	

								Lower limit	Upper limit
Step 1^a^	Female age	-.033	.027	1.513	1	.219	.967	.918	1.020
	Gn/egg	.000	.000	.354	1	.552	1.000	.999	1.001
	LH level on hCG injection day	-.105	.052	4.051	1	.044	.900	.813	.997
	P level on hCG injection day	.052	.057	.816	1	.366	1.053	.941	1.178
	Number of follicles with a diameter of ≥14 mm	-.062	.041	2.240	1	.134	.940	.867	1.019
	Sperms/egg	-.097	.079	1.534	1	.216	.907	.778	1.058
	Total number of eggs	.032	.028	1.298	1	.255	1.032	.977	1.090
	E2 level on hCG injection day	.000	.000	.222	1	.638	1.000	1.000	1.000
	Constant	.931	1.003	.861	1	.354	2.536		

**Table-III:** Comparison of embryonic development between the two groups.

Items	Group A(n=347)	Group B(n=189)	P
Good-quality embryo rate on D3	(24.31) 476/1958	(23.05) 213/924	0.460
Blastocyst formation rate	(44.18) 680/1539	(45.13) 338/749	0.670
Available embryo rate	(57.97) 1135/1958	(61.80) 571/924	0.051
Egg utilization rate	(38.87) 1135/2920	(29.60) 571/1929	0.000

## DISCUSSION

However, no difference in the average number of retrieved eggs was observed between the two groups in the present study, which was probably explained by different grouping methods and aspects of concern. Due to the possible influencing factors of abnormal fertilization in patients with primary infertility and the controversial influence of short-term fertilization combined with early cumulus cell removal on fertilization^7^,^8^, cycles for secondary infertility with antagonist protocol and long-term fertilization were selected in the present study to avoid the effect of different protocols on the homogeneity of eggs. The results showed that female age, levels of P and E2 on hCG injection day, number of follicles with a diameter of ≥14 mm on hCG injection day, and total number of eggs retrieved were not independent influencing factors for polyspermy.

Meanwhile, the effects of laboratory fertilization steps on the development of polyspermy were analyzed, and the results showed that sperm concentration and fertilization concentration (x10,000 sperms/egg) were not influencing factors for polyspermy, which was similar to the results of Turathum B et al.^9^-^11^, i.e., the level of LH on hCG injection day was an influencing factor for polyspermy. However, number of eggs and number of follicles with a diameter of ≥14 mm on hCG injection day were not included in the equation, and the main reasons included that only cycles with a high proportion of abnormal fertilization, i.e., the ratio of multiple PN: 2PN ≥20%, were used for grouping in the present study, and those with a low abnormal fertilization rate are not included. In addition, by using this cutoff value in grouping, cycles with a poor fertilization rate, which might be ignored due to the low incidence of polyspermy, were included and analyzed in the present study.

The incidence of polyspermy during conventional IVF is 2%-10%.^12^,^13^ Numerous studies on the influencing factors of polyspermy, including sperm concentration, egg quality, zona pellucida, fertilization conditions, and abnormally high hormone levels, have been reported, and controversies on this topic, however, still exist.^14^-^19^ Inconsistent findings of different studies are mainly explained by complex factors such as the experimental methods and the criteria for grouping. For instance, The mainstream ovulation induction protocols used in the IVF center may vary, patient factors, such as primary and secondary infertility, may exist, and different fertilization methods, including long-term fertilization, short-term fertilization, and short-term fertilization combined with early cumulus cell removal, are used.

Furthermore, the process of sperm-egg fertilization is regulated by multiple mechanisms. The ovulation induction process may affect the quality of eggs, which directly affects the zona pellucida reaction and cortical reaction during fertilization. Studies have suggested that polyspermy usually occurs in patients when a large number of eggs are retrieved with diverse development levels. The cortical and zona pellucida reactions are not complete in immature eggs, which may lead to an increased incidence of polyspermy.^20^ In addition, embryo outcomes of the two groups were analyzed. No differences in the good-quality embryo rate on D3 and blastocyst formation rate were found, and the available embryo rate was better in the polyspermy group. However, the egg utilization rate was significantly decreased in the polyspermy group compared with that in the control group, and the difference was statistically significant. Egg utilization rate can most directly reflect the embryo outcomes of the treatment cycles, and thus the entire treatment cycle was affected by polyspermy.

### Limitations:

However, this study also has some shortcomings, ovulation induction is a very complex process, and homogeneous outcomes may not be achieved in individualized ovulation induction. Big data analysis is required for the determination of the time and dose of LH administration, as well as the level of LH appropriate for egg development. It is difficult to fully explain the related mechanisms only based on the aspects investigated in the present study.

## CONCLUSIONS

In the process of ovulation induction in antagonist protocol, the appropriate addition of LH can improve embryo outcomes. The possible factors in clinical and laboratory settings that may increase the incidence of polyspermy were analyzed, which is helpful to avoid these risks and guide clinical treatment.

### Authors’ Contributions:

**HS** and **QS:** Concept and desing**n,** Carried out the studies, participated in collecting data, and drafted the manuscript and are responsible and accountable for the accuracy or integrity of the work.

**JL,CL and** **RL**: Literature search**,** Performed the statistical analysis and participated in its design.

All authors read and approved the final manuscript.
